# The Impact of Longer Biliopancreatic Limb Length on Weight Loss and Comorbidity Improvement at 5 Years After Primary Roux-en-Y Gastric Bypass Surgery: A Population-Based Matched Cohort Study

**DOI:** 10.1007/s11695-024-07267-5

**Published:** 2024-07-09

**Authors:** Floris F. E. Bruinsma, Simon W. Nienhuijs, Ronald S. L. Liem, Jan Willem M. Greve, Perla J. Marang-van de Mheen, on behalf of the Dutch Audit for Treatment of Obesity Research Group, G. J. D. van Acker, J. Apers, L. M. de Brauw, S. M. M. de Castro, S. L. Damen, I. F. Faneyte, G. van’ t Hof, F. H. W. Jonker, R. A. Klaassen, E. A. G. L. Lagae, B. S. Langenhoff, R. S. L. Liem, A. A. P. M. Luijten, S. W. Nienhuijs, R. M. Smeenk, S. J. M. Smeets, W. Vening, M. J. Wiezer, E. de Witte

**Affiliations:** 1 Department of Surgery, Maastricht University Medical Centre, NUTRIM School for Nutrition and Translational Research in Metabolism, P. Debyelaan 25, 6229 HX Maastricht, The Netherlands; 2 Scientific Bureau, Dutch Institute for Clinical Auditing, Leiden, The Netherlands; 3 Department of Surgery, Catharina Hospital, Eindhoven, The Netherlands; 4 Department of Surgery, Groene Hart Hospital, Gouda, The Netherlands; 5 Nederlandse Obesitas Kliniek, Gouda and The Hague, Gouda, The Netherlands; 6 Department of Surgery, Zuyderland Medical Centre, Heerlen, The Netherlands; 7 Nederlandse Obesitas Kliniek, Heerlen, The Netherlands; 8 Department of Biomedical Data Sciences, Medical Decision Making, Leiden University Medical Centre, Leiden, The Netherlands; 9 Safety & Security Science, Faculty of Technology, Policy and Management, Delft University of Technology, Delft, The Netherlands

**Keywords:** Roux-en-Y gastric bypass, Biliopancreatic limb length, Population-based, Propensity score matching, Comorbidity resolution

## Abstract

**Introduction:**

Different limb lengths are used in Roux-en-Y gastric bypass (RYGB) surgery, as there is no consensus which limb length strategy has the best outcomes. The biliopancreatic limb (BPL) is thought to play an important role in achieving weight loss and associated comorbidity resolution. The objective of this study was to assess the impact of a longer BPL on weight loss and comorbidity improvement at 5 years after primary RYGB.

**Methods:**

All patients aged ≥ 18 years undergoing primary RYGB between 2014–2017 with registered follow-up 5 years after surgery were included. Long BPL was defined as BPL ≥ 100 cm and short BPL as BPL < 100 cm. The primary outcome was achieving at least 25% total weight loss (TWL) at 5 years. Secondary outcomes included absolute %TWL and improvement of comorbidities. A propensity score matched logistic and linear regression was used to estimate the difference in outcomes between patients with long and short BPL.

**Results:**

At 5 years, long BPL had higher odds to achieve ≥ 25% TWL (odds ratio (OR) 1.19, 95% confidence interval (CI) [1.01 – 1.41]) and was associated with 1.26% higher absolute TWL (β = 1.26, 95% CI [0.53 – 1.99]). Furthermore, long BPL was more likely to result in improved diabetes mellitus (OR = 2.17, 95% CI [1.31 – 3.60]) and hypertension (OR = 1.45, 95% CI [1.06 – 1.99]).

**Conclusion:**

Patients undergoing RYGB with longer BPL achieved higher weight loss and were more likely to achieve improvement of comorbidities at 5 years.

**Graphical Abstract:**

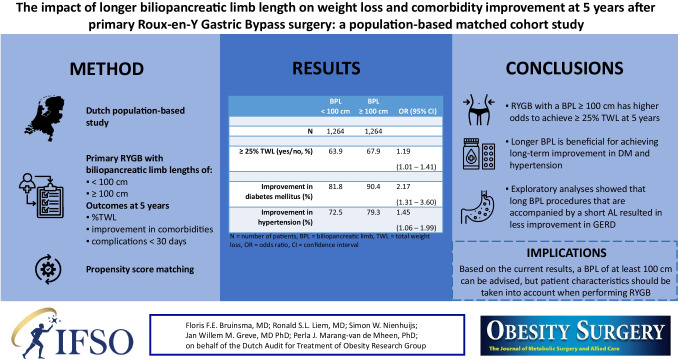

**Supplementary Information:**

The online version contains supplementary material available at 10.1007/s11695-024-07267-5.

## Introduction

Roux-en-Y gastric bypass (RYGB) is one of the most frequently performed procedures in metabolic and bariatric surgery (MBS) [1] and results in three intestinal limbs: the biliopancreatic limb (BPL), the alimentary limb (AL), and the common channel (CC). Measuring only BPL and AL seems to have gained satisfactory results without any hazard or time loss when mobilizing and measuring CC; therefore, CC is generally not measured [2]. As there are no criteria regarding the optimal length of the BPL and AL, many variations of the RYGB procedure with various combinations of limb lengths exist. There is ongoing debate on which limb length combination results in the best outcome.

Previous studies have found that elongation of the AL had no or little effect on weight loss [3–5], which suggests that nutrient uptake also takes place in the AL and may explain why the focus of research shifted towards the BPL. Although multiple prospective trials found that a longer BPL induced extra weight loss [6–8], a recent meta-analysis of 10 randomized controlled trials (RCTs) directly comparing different BPL length strategies, showed no short- and long-term differences in weight loss (12 months and 48–72 months, respectively) [9]. Only at 24 months, higher weight loss was present in the long BPL group, so that the authors concluded that this isolated finding was not clinically relevant. Even though evidence from such a meta-analysis of RCTs constitutes the highest level of evidence due to the randomization of patients, the patients included in those trials are mostly a selection that do not necessarily reflect those treated in daily clinical practice.

Although elongation of the BPL might result in more micro nutritional deficiencies [10, 11], other impacts of a longer BPL appear to be in its favor. Besides the possible effect on weight loss, there are indications that comorbidity resolution might be influenced by elongation of the BPL as well. Of the various obesity-related comorbidities, particularly type 2 diabetes (T2D) has been investigated. A recently published meta-analysis examined T2D improvement rates for different limb length combinations in RYGB and found in meta-regression analysis that BPL ≥ 100 cm was associated with higher T2D improvement rates than BPL < 100 cm, while such an association was not found for the AL [12]. Hence, it led to the conclusion that particularly BPL length is involved in the underlying mechanisms of metabolic improvement after RYGB. However, this meta-regression analysis had some limitations, including the inability to correct for patient characteristics and the predominance of studies that did not directly compare different limb length strategies. Only few studies have directly compared BPL length strategies on comorbidity resolution [13–16], with only one being a RCT showing no significant difference in HbA1C improvement at 12 months [16]. This underlines the importance of additional research examining the impact on comorbidities, in particular for long-term outcomes in an unselected patient population.

Therefore, this study used a population-based cohort from a nationwide quality registry to compare patients undergoing RYGB with a long BPL versus a short BPL on achieved weight loss and comorbidity improvement at 5 years follow-up. Propensity score matching (PSM) was used to correct for confounding by indication, to obtain reliable estimates of the treatment effect similar to those from RCTs while using real-world data including all patients treated in daily clinical practice [17].

## Methods

### Study Design and Setting

A population-based cohort was derived from the Dutch Audit for Treatment of Obesity (DATO). DATO is a mandatory nationwide quality registry of MBS that includes all bariatric procedures from 2014 onwards [18]. All Dutch hospitals performing MBS participate in the registry and register data on patient characteristics, procedures, complications, weight loss, and comorbidity reduction at annual follow-up periods up to 5 years thereby including all patients undergoing MBS in the Netherlands. On-site data verification has proven high validity of the data [19]. In DATO, all annual follow-up periods have an approximation of ± 3 months, meaning that a follow-up between 9–15 months is considered a 1-year follow-up moment, follow-up between 21–27 months as a 2-year follow-up moment, and so on. In that context, the 5-year follow-up is defined as any follow-up period between 57 and 63 months.

This study was approved by the scientific committee of DATO and by the privacy committee of the Dutch Institute for Clinical Auditing (DICA) and has been performed following the ethical standards stated in Dutch law. In accordance with the DICA regulations, no informed consent from patients was required as it concerns an opt-out registry.

### Patients and Definitions

Before selecting patients, data were examined on unlikely values which were recoded as missing values, for example if a patient received primary bariatric surgery while body mass index (BMI) was 21 kg/m^2^, or when American Society of Anesthesiologist (ASA) score was 5 (moribund patients are very unlikely to undergo primary bariatric surgery). Criteria used for data-cleaning can be found in the appendix.

All patients aged ≥ 18 years undergoing primary RYGB between 2014–2017 were included if they had a registered 5-year follow-up visit with weight recorded. Patients were excluded if they had missing baseline characteristics (i.e. age, BMI, sex, ASA score), procedure characteristics (i.e. procedure type (no banded RYGB procedures), BPL and AL lengths), or comorbidity status (i.e. presence of diabetes mellitus (DM), hypertension, dyslipidemia, obstructive sleep apnea syndrome (OSAS), gastro-esophageal reflux disease (GERD) and musculoskeletal pain).

**Fig. 1:**
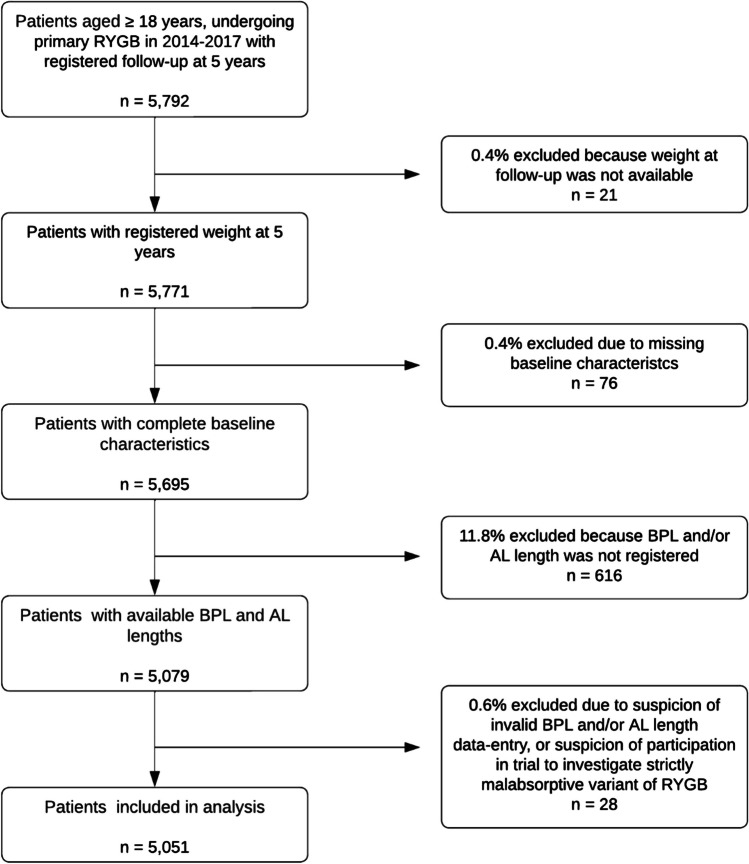
Flowchart of patient inclusion. RYGB = Roux-en-Y gastric bypass, n = number, BPL = biliopancreatic limb, AL = alimentary limb

*Long BPL* was defined as BPL ≥ 100 cm and *short BPL* as BPL < 100 cm, consistent with a previous meta-analysis [12], and because in clinical practice Dutch surgeons typically perform primary RYGB with BPL lengths either reasonably longer or shorter than 100 cm. BPL and AL lengths < 40 cm or > 250 cm were considered invalid data-entries, as in Dutch daily practice it is very unlikely that those lengths would reflect the true limb lengths. Some patients registered in DATO possibly participated in trials and therefore could have had very long AL (> 250 cm) [20], but these procedures have the risk of resulting in a very short common channel, thereby inducing a lot of malabsorption. Therefore, these procedures were considered malabsorptive and not regular RYGB procedures and were therefore excluded from the analysis [21, 22]. Fig. 1 shows that only a small number of patients (n = 28, 0.6%) were excluded due to these limb length criteria.

## Outcomes

The primary outcome was achieving 25% total weight loss (25% TWL, yes/no) at 5 years after surgery. Although 20% TWL is a widely used criterium for defining a successful outcome, it is a conservative threshold with the largest part of patients achieving this result [23], whereas 25% TWL is more discriminative and therefore should be considered as a better cut-off for good response [24]. Percentage total weight loss (%TWL) is defined as [preoperative weight – follow-up weight] / preoperative weight * 100. Secondary outcomes were %TWL on a continuous scale, absolute change in BMI (ΔBMI), percentage excess weight loss (%EWL, calculated as ΔBMI / [BMI at screening – 25] * 100), postoperative complications and any improvement (i.e. complete or partial resolution, or complete or partial relieve of symptoms) of the comorbidities DM, hypertension, dyslipidemia, OSAS, GERD and musculoskeletal pain at 5 years follow-up. Partial resolution is defined as requiring a lower dose of comorbidity-specific medication or improved clinical tests, depending on the comorbidity. Exact definitions of comorbidity diagnosis, improvement and resolution criteria during follow-up are described elsewhere [25]. Only postoperative complications within 30 days of surgery with a score of ≥ 3 on the Clavien-Dindo scale were taken into consideration [26].

### Statistical Analyses

First, differences in baseline characteristics between the long and short BPL group were analyzed using chi-square tests for categorical variables and the t-test or Mann–Whitney U test for continuous variables, depending on the distribution. To correct for confounding by indication, PSM was performed to create balanced groups of long and short BPL procedures [17]. All baseline characteristics and comorbidities mentioned above were used to calculate the propensity scores. 1:1 PSM was conducted using the nearest neighbor method with a caliper width of 0.2, using the standard deviation of the logit of the propensity score, as recommended in existing literature [27, 28]. A standardized mean difference (SMD) of < 0.10 was considered to indicate balanced groups.

Matched groups were then compared on the primary and secondary outcomes, using logistic regression analysis for dichotomous outcomes and linear regression for continuous outcomes, including the treatment group and the propensity score as independent variables. In case of imbalance on any of the baseline characteristics (i.e. SMD ≥ 0.10), this variable was included in the regression analysis to adjust for this imbalance [17]. Improvement of comorbidities was analyzed in the subgroup of patients with a specific comorbidity at baseline. An alpha of < 0.05 was considered as a statistically significant difference in all analyses. All statistical analyses were conducted using RStudio version 2022.07.1 (R Foundation for Statistical Computing, Vienna, Austria).

### Sensitivity Analysis

As most studies report the effect of longer BPL on short-term results, the same analyses were performed while also including patients with a shorter follow-up. All patients who received primary RYGB between 2014–2021 with at least 1 year follow-up were analyzed, using the weight loss or comorbidity status at the last available follow-up moment as the dependent variable, meaning that if a patient for example had recorded follow-up at 1, 2, and 3 years, only the outcomes at 3 years were used for this analysis. In this way, a larger cohort of patients is included e.g. also patients with the last follow-up at 3 or 4 years, resulting in higher power, particularly for the analyses in the subgroup of patients with comorbidities. PSM was performed in the same way as described above, but with exact matching on the follow-up moment to ensure that patients with e.g. 4 year follow-up were matched to others at the same follow-up moment, rather than for instance with the outcomes of a patient at 1-year follow-up.

For both the primary and the sensitivity analysis we explored whether the AL length influenced the outcomes, by creating subgroups with long or short AL (long AL defined as > 100 cm and short AL as ≤ 100 cm). As only very few patients received short BPL combined with short AL, the short BPL group was not further subdivided.

## Results

In total, 5,792 patients undergoing primary RYGB had a registered follow-up at 5 years. After applying the exclusion criteria 5,051 patients (87.2%) were included in the analysis, as shown in Fig. 1. Table 1 shows that before matching considerable differences were present in most baseline characteristics. After matching, groups were well-balanced with 1,264 patients in both the long and short BPL group. Median BPL length was 150 cm in the long BPL group (inter-quartile range (IQR) 150 – 150) and 70 cm in the short BPL group (IQR 50 – 75), which shows the variation within groups and confirms that the chosen cut-off differentiates between longer and shorter BPL. Median AL length was 100 cm in the long BPL group (IQR 75–100) and 150 cm in the short BPL group (IQR 150–150). Median BPL and AL lengths with their IQRs were the same before and after matching.

**Table 1:** Baseline characteristics of patients with 5-year follow-up data available, before and after propensity score matching

	Before matching		P-value	SMD	After matching		SMD
	BPL < 100 cm	BPL ≥ 100 cm			BPL < 100 cm	BPL ≥ 100 cm	
n	3,780	1,272			1,264	1,264	
Age (median (range))	46 [18, 69]	48 [18, 69]	< 0.01	0.21	48 [19, 69]	48 [18, 69]	0.02
BMI (median (range)), kg/m^2^	41.7 [31.9, 75.9]	42.4 [31.0, 66.7]	< 0.01	0.18	42.5 [32.0, 72.5]	42.4 [31.0, 66.7]	0.01
Sex (n, %)							
male	632 (16.7)	221 (17.4)	0.62	0.02	233 (18.4)	219 (17.3)	0.03
female	3148 (83.3)	1051 (82.6)			1031 (81.6)	1045 (82.7)	
ASA-score (n, %)							
I	90 (2.4)	53 (4.2)	< 0.01	0.24	58 (4.6)	52 (4.1)	0.04
II	2700 (71.4)	959 (75.4)			941 (74.4)	959 (75.9)	
III	971 (25.7)	234 (18.4)			248 (19.6)	234 (18.5)	
IV	5 (0.1)	5 (0.4)			4 (0.3)	4 (0.3)	
‘Unknown’	14 (0.4)	21 (1.7)			13 (1.0)	15 (1.2)	
Diabetes mellitus (n, %)							
* Not present*	2906 (76.9)	934 (73.4)	< 0.01	0.12	926 (73.3)	932 (73.7)	0.02
* Present without medication*	324 (8.6)	156 (12.3)			148 (11.7)	151 (11.9)	
* Present with medication*	550 (14.6)	182 (14.3)			190 (15.0)	181 (14.3)	
Hypertension (n, %)							
* Not present*	2342 (62.0)	721 (56.7)	< 0.01	0.11	725 (57.4)	717 (56.7)	0.01
* Present without medication*	613 (16.2)	234 (18.4)			228 (18.0)	230 (18.2)	
* Present with medication*	825 (21.8)	317 (24.9)			311 (24.6)	317 (25.1)	
Dyslipidemia (n, %)							
* Not present*	2896 (76.6)	972 (76.4)	0.77	0.02	991 (78.4)	968 (76.6)	0.04
* Present without medication*	486 (12.9)	172 (13.5)			156 (12.3)	168 (13.3)	
* Present with medication*	398 (10.5)	128 (10.1)			117 (9.3)	128 (10.1)	
OSAS (n, %)							
*Not present*	3010 (79.6)	1063 (83.6)	< 0.01	0.16	1035 (81.9)	1055 (83.5)	0.04
*Present without therapy*	409 (10.8)	141 (11.1)			158 (12.5)	141 (11.2)	
*Present with therapy*	361 (9.6)	68 (5.3)			71 (5.6)	68 (5.4)	
GERD (n, %)							
* Not present*	3365 (89.0)	1107 (87.0)	< 0.01	0.17	1115 (88.2)	1101 (87.1)	0.03
* Present without medication*	169 (4.5)	33 (2.6)			30 (2.4)	33 (2.6)	
* Present with medication*	246 (6.5)	132 (10.4)			119 (9.4)	130 (10.3)	
Musculoskeletal pain (n, %)							
* Not present*	1904 (50.4)	560 (44.0)	< 0.01	0.13	549 (43.4)	557 (44.1)	0.01
* Present*	1876 (49.6)	712 (56.0)			715 (56.6)	707 (55.9)	
BPL length (median [IQR]), cm	70 [50 – 75]	150 [150 – 150]			70 [50 – 75]	150 [150 – 150]	
AL length (median [IQR]), cm	150 [150 – 150]	100 [75 – 100]			150 [150 – 150]	100 [75 – 100]	

*SMD* standardized mean difference, *n* number, *BPL* biliopancreatic limb, *IQR* inter-quartile range, *cm* centimeter, *AL* alimentary limb, *SD* standard deviation, *ASA* American Society of Anaesthesiologists, *OSAS* obstructive sleep apnea syndrome, *GERD* gastro-esophageal reflux disease

At 5 years follow-up, 65.9% of the matched patients achieved at least 25% TWL. However, the odds to achieve 25% TWL was significantly higher for patients in the long BPL group compared with those receiving a shorter BPL (odds ratio (OR) 1.19, 95% CI [1.01 – 1.41], P = 0.04, see Table 2). Considering %TWL on a continuous scale, this was 1.26%-points higher in the long BPL cohort (29.7% vs. 28.4%, β = 1.26, 95% CI [0.53 – 1.99], P < 0.01), and %EWL and ΔBMI were also significantly higher.

**Table 2:** Primary and secondary outcomes for long and short BPL at 5 years in matched patients

Weight loss outcomes	Weight loss at 5 years			OR (95% CI)	β *(95% CI)*	*P*-value
	N = 2,528	Short BPL (< 100 cm) N = 1,264	Long BPL (≥ 100 cm) N = 1,264			
≥ *25% TWL (yes/no, %)*		63.9	67.9	1.19 (1.01 – 1.41)		0.04
*%TWL (%)*		28.4	29.7		1.26 (0.53 – 1.99)	< 0.001
*%EWL (%)*		69.9	73.2		3.29 (1.45 – 5.13)	< 0.001
*Δ BMI (kg/m*^*2*^*)*		12.5	13.1		0.55 (0.19 – 0.91)	0.003
Comorbidity	Improvement at 5 years (%)					
	*n*	Short BPL (< 100 cm)	Long BPL (≥ 100 cm)			
*Diabetes mellitus*	*659*	81.8	90.4	2.17 (1.31 – 3.60)		0.002
*Hypertension*	*1,059*	72.5	79.3	1.45 (1.06 – 1.99)		0.02
*Dyslipidemia*	*578*	73.6	74.2	1.02 (0.66 – 1.56)		0.94
*OSAS*	*423*	85.4	92.1	2.00 (0.94 – 4.26)		0.07
*GERD*	*315*	90.0	78.6	0.37 (0.13 – 1.12)		0.08
*Musculoskeletal pain*	*1,425*	60.3	58.1	0.92 (0.66 – 1.27)		0.60

Reference = short BPL

β reflects the absolute difference between short and long BPL

*N* number of patients, *OR* odds ratio, *CI* confidence interval, *BPL* biliopancreatic limb, *TWL* total weight loss, *EWL* excess weight loss, *ΔBMI* absolute change in body mass index, *n* number of patients with the comorbidity at baseline, *OSAS* obstructive sleep apnea syndrome, *GERD* gastro-esophageal reflux disease

With respect to comorbidity resolution, patients with a long BPL were more likely to show improvement in DM (OR = 2.17, 95% CI [1.31 – 3.60], P < 0.01) and hypertension (OR = 1.45, 95% CI [1.06 – 1.99], P = 0.02). There were no significant differences in improvement of the other comorbidities, as also shown in Table 2. These results should be interpreted in the context of data completeness of comorbidity status during follow-up, which was over 80% for DM but relatively low for the other comorbidities as presented in supplementary Table 1. Patients receiving long or short BPL had similar risks of postoperative complications (OR = 1.11, 95% CI [0.67 – 1.84], P = 0.70).

Creating subgroups of long and short AL length within the long BPL group, showed that particularly patients receiving a longer BPL (median 150 cm) combined with a short AL had significantly higher odds to achieve improvement in diabetes and hypertension at 5 years, but also significantly lower odds to achieve improvement in GERD, as shown in supplementary Table 3. Patients receiving long BPL (median 100 cm) combined with a long AL did not differ in outcomes at 5 years from patients receiving a short BPL.

### Sensitivity Analysis

In total, 32,070 patients had at least 1-year follow-up data available and after applying the exclusion criteria 28,553 (89.0%) patients were eligible for analysis, with 13,258 patients receiving long BPL and 15,295 patients short BPL (supplementary Table 2). The mean follow-up period for patients who received long BPL was 2.39 years (median 2.0; IQR 1.0 – 3.0) and 2.76 years for patients who received short BPL (median 3.0; IQR 1.0 – 4.0), indicating a slight shift towards longer BPL procedures in more recent years. Matching resulted in well-balanced groups, including 11,518 patients in each group. The mean follow-up in the matched cohort was 2.48 years (median 2.0; IQR 1.0 – 4.0).

The odds to achieve 25% TWL at the last available follow-up was significantly higher for patients receiving a long BPL (OR = 1.23, 95% CI [1.15 – 1.31], P < 0.01). The other weight-related outcomes also showed similar significant differences as in the primary analysis. Long BPL had favorable results on improvement of DM, hypertension, dyslipidemia, and OSAS, as shown in Table 3. In contrast, patients receiving long BPL had lower odds to achieve improvement in GERD (OR = 0.65, 95% CI [0.49 – 0.85], P < 0.01). There were no differences for amelioration of musculoskeletal pain or risk of postoperative complications.

**Table 3:** Primary and secondary outcomes for long and short BPL in matched patients with at least 1 year follow-up

Weight loss outcomes	Weight loss at 1–5 years						
	*N* = 23,036		Short BPL (< 100 cm) N = 11,518	Long BPL (≥ 100 cm) N = 11,518	OR (95% CI)	β (95% CI)	P-value
≥ *25% TWL (yes/no, %)*			77.0	80.5	1.23 (1.15–1.31)		< 0.001
*%TWL (%)*			31.2	32.2		1.03 (0.80 – 1.25)	< 0.001
*%EWL (%)*			78.9	81.5		2.64 (2.02 – 3.25)	< 0.001
*Δ BMI (kg/m*^*2*^*)*			12.8	13.3		0.48 (0.37 – 0.59)	< 0.001
	Improvement at 1–5 years (%)						
Comorbidity	*n*	*Comorbidity status available at last FU (%)*	Short BPL (< 100 cm)	Long BPL (≥ 100 cm)			
*Diabetes mellitus*	4,747	80.2	85.6	92.9	2.17 (1.75 – 2.69)		< 0.001
*Hypertension*	8,490	80.3	75.4	80.0	1.31 (1.16 – 1.46)		< 0.001
*Dyslipidemia*	5,079	74.9	69.0	73.0	1.22 (1.06 – 1.41)		0.006
*OSAS*	3,971	74.0	82.0	89.8	1.90 (1.54 – 2.36)		< 0.001
*GERD*	4,195	43.4	88.8	83.5	0.65 (0.49 – 0.85)		0.002
*Musculoskeletal pain*	10,549	62.3	72.0	72.3	1.03 (0.92 – 1.14)		0.65
Adverse events							
*CD3* + < *30 days (%)*			2.27	1.96	0.86 (0.72 – 1.03)		0.11

Reference = short BPL

β reflects the absolute difference between short and long BPL

*N* number of patients, OR = odds ratio, CI = confidence interval, BPL = biliopancreatic limb, TWL = total weight loss, EWL = excess weight loss, ΔBMI = absolute change in body mass index, n = number of patients with the comorbidity at baseline, FU = follow-up, OSAS = obstructive sleep apnea syndrome, GERD = gastro-esophageal reflux disease, CD = Clavien-Dindo

Propensity score matching was done using the nearest neighbor method with exact matching on the last available follow-up moment

The favorable results for the long BPL group were found in both AL length subgroups for achieving 25% TWL and improvement in DM and OSAS, but with respect to improvement in hypertension only for patients receiving a longer BPL (median 150 cm) and short AL and with respect to improvement in dyslipidemia and musculoskeletal pain only for patients receiving long BPL (median 100 cm) and long AL. (supplementary Table 4). The lower odds to achieve improvement in GERD was only found for patients receiving a longer BPL and short AL.

## Discussion

This propensity score matched, nationwide analysis showed that at 5 years after primary RYGB, patients with a BPL ≥ 100 cm had higher odds to achieve 25% TWL than patients receiving a shorter BPL. Higher absolute %TWL, %EWL and ΔBMI were found as well. Furthermore, patients with a long BPL were more likely to achieve improvement in DM and hypertension. The sensitivity analysis including also patients with shorter follow-up duration showed similar results, that a longer BPL was beneficial for all weight loss parameters as well as improving the comorbidities DM, hypertension, dyslipidemia, and OSAS. In contrast, long BPL had less favorable results with respect to GERD improvement, which was found to be associated with shorter AL length.

These results add to the existing body of literature where results do not consistently point in the same direction. Although multiple prospective trials found that a longer BPL enhanced weight loss [6, 8, 11], a recent meta-analysis that identified 10 RCTs comparing different BPL lengths in RYGB found no difference in long-term weight loss [9]. Since RCTs often contain selected patients due to strict selection criteria, this may partly explain the difference in results so that when all patients from daily surgery practice are included, there is a difference in the likelihood to achieve ≥ 25% TWL at 5 years between longer and shorter BPL. This could similarly have played a role in former research, particularly when absolute weight loss differences are small, that some studies found a difference in weight loss, and others did not. Because weight loss differences are small, evaluating the differences in comorbidity resolution are of increased relevance.

The current study found significantly higher odds of DM improvement at 5 years for patients receiving long BPL, which is in line with the findings in a recent meta-analysis [12]. However, this meta-analysis mainly included studies not directly comparing different BPL length strategies and only looked at results one year after surgery, so that the current study adds to existing literature that there is a benefit when directly compared and at long-term follow-up. The mechanisms underlying this advantage of a long BPL are not entirely clear and should be explored in future research. Current literature thus far shows that gastric bypass procedures induce resolution of DM more than would be expected from the effect of weight loss alone [29]. This most likely relies on adaptations in gut hormone secretion by the altered passage of ingested food, upregulating the secretion of hormones with insulinotropic effects, such as glucagon-like peptide 1 (GLP1) and peptide YY [29–31]. Therefore, additional research on whether elongation of the BPL affects this alteration in gut hormones would be valuable. Furthermore, the current study showed that patients receiving a long BPL were also more likely to achieve improvement in hypertension. Multiple prospective trials comparing different limb length strategies in the short and long-term have investigated hypertension as secondary outcome but found no benefit when BPL was longer [6, 8, 32, 33]. However, because these studies were primarily aimed at detecting differences in weight loss, they were likely underpowered to detect differences in hypertension resolution. Having included over 1,000 patients with hypertension in our primary analysis, this may be one of the reasons why it was possible to detect the beneficial impact of a longer BPL on long-term hypertension improvement. Still, research on the underlying mechanisms for this observed effect is needed. As it becomes clearer that alterations in gut hormones, but also changes in bile acid concentrations and microbiome play a key role in gastric bypass surgery [29, 34–37], additional research on the extent to which the BPL length influences these alterations, and if these alterations influence metabolic outcomes, would be relevant.

Having included over 23,000 patients with also shorter follow-up in the sensitivity analysis, it showed similar results and estimates as in the primary analysis. However, the impact on a larger number of comorbidities reached statistical significance, i.e. also for improvement of dyslipidemia and OSAS, likely because more patients were included in the sensitivity analysis so that this analysis had more power to detect these differences. This is supported by the fact that the point estimates were similar but had smaller confidence intervals, so that the difference is similar but becomes significant because of the higher number of included patients.

The sensitivity analysis also showed that long BPL had significantly lower odds to achieve improvement of GERD, which to our knowledge has not been described previously. A possible explanation may be that complaints of (acidic) reflux are substituted to some extent by biliary reflux, as an open connection between the gastric pouch and jejunum is created in RYGB procedures. Performing short BPL RYGB in general means creating a long AL, as also shown in the results of the current study, therefore creating a longer route for bile to travel before arriving at the esophagus, possibly reducing the risk of biliary reflux. The results of the exploratory analysis support this theory, as short AL procedures were related to having lower odds to achieve improvement in GERD. Bile reflux after RYGB has been described before [38, 39], with one study reporting on 16 patients with bile in their gastric pouch and finding the AL to be very short during revisional surgery (mean 37 cm, range 20 – 62 cm). Lengthening the AL to 100 cm eliminated symptoms in all cases, which supports the above theory.

Even though all weight loss outcomes were statistically different, its clinical relevance is up for debate. The absolute difference in %TWL of 1.3 percentage points translates to a difference in achieved weight loss of approximately 1–2 kg, which seems marginal. However, except for the lower odds of achieving GERD improvement, no disadvantages of a long BPL were found. In fact, long BPL predominantly had better outcomes compared with short BPL, most outspokenly so in terms of DM improvement. It therefore seems reasonable to recommend that patients should receive a BPL ≥ 100 cm, as this strategy seems most beneficial. A longer AL should accompany the procedure for patients suffering from GERD at baseline. Despite that mainly advantages of a longer BPL were found, it should be kept in mind that the current study did not evaluate long-term complications such as internal herniation and nutritional deficiencies [40]. Particularly for the latter, there are indications that longer BPL might be unfavorable [11, 41, 42]. Elongation of the BPL results in a shorter total alimentary limb length (TALL, AL + common channel), which is responsible for nutrient uptake. However, this appears to be specifically important for procedures with very long BPL lengths, such as (variations of) the biliopancreatic diversion, where TALL often remains insufficient to absorb enough (micro-) nutrients [21, 43]. Since the median BPL length in the long BPL cohort of the current study was 150 cm, it seems likely that TALL remained sufficient for adequate nutrient uptake [2, 21], provided that patients adhere to the dietary advice and prescribed supplements.

Strengths of this study include that to our knowledge, this was the first population-based study evaluating the impact of longer BPL in RYGB that used PSM to correct for confounding by indication to produce high-quality evidence, while also including unselected patients reflecting daily surgery practice. The large cohort of patients combined with long-term results therefore adds strong evidence for any differences that seem to exist between long and short BPL in RYGB procedures. Nevertheless, some limitations should be noted. PSM can only correct for measured confounders and consequently this study was not able to correct for unmeasured confounders such as disease duration, socioeconomic status, and peri-operative guidance (e.g. participation in a prehabilitation program or consultations with a dietitian). The implicit assumption therefore is that such characteristics become evenly distributed by the pseudo-randomization of PSM. In addition, the number of patients with complete follow-up data at 5 years may be a selection. Data completeness was highest for patients with DM with > 80%, indicating that selection bias is less of a concern for this outcome, but could be an issue for the other comorbidities with low data completeness. In the Netherlands, when comorbidities are well-regulated after MBS, treatment and monitoring of these comorbidities is often done by the general practitioner. This can result in suboptimal registration of the comorbidity status during follow-up. However, even with relatively low completeness, the key issue is whether this was evenly distributed between the long and short BPL groups for it to induce bias. Since differences in completeness were small, any bias that may result from this is expected to be minimal. Finally, the current study did not report on complications beyond 30 days, such as nutritional deficiencies, chronic diarrhea, and internal herniation, which are relevant for the decision to employ a long or short BPL and should be investigated in future research.

## Conclusion

RYGB with a BPL ≥ 100 cm had higher odds to achieve ≥ 25% TWL at 5 years. Beneficial effects of long BPL were also found for achieving long-term improvement in DM and hypertension. At the same time, short AL procedures were related to worse outcomes in terms of GERD improvement. This underlines that patient characteristics should be taken into consideration in decision-making on limb length strategies in RYGB. Based on the current results, a BPL of ≥ 100 cm can be advised, but it should be kept in mind that long BPL procedures are frequently accompanied by a shorter AL, which can be undesirable for patients with GERD. Therefore, for these patients, it could be preferable to receive a longer AL, potentially at the cost of a somewhat shorter BPL.

### Electronic supplementary material

Below is the link to the electronic supplementary material.

Supplementary file1 (DOCX 29 KB)

## Appendix

### Criteria Used for Preliminary Data-Cleaning

Age < 18 or > 80 was translated to ‘NA’.

Baseline weight < 70 or ≥ 450 was translated to ‘NA’.

Baseline BMI < 30 kg/m^2^ was translated to ‘NA’.

ASA score documented as 5 was considered invalid and translated to ‘NA’.

Follow-up weight < 40 or ≥ 450 was translated to ‘NA’.

When comorbidity status was documented as ‘remission/partial remission/equal/worse’, but patient was not registered to be having the comorbidity preoperatively, the comorbidity status during follow-up was translated to ‘NA’.

Two patients were extracted because of untrustworthy weight values. One patient went without intermitting surgery from 107 kg on the 4-year follow-up moment to 171 kg at the 5-year follow-up moment. One patient had a screening weight of 331 kg while her preoperative weight was 135 kg, making it suspicious for erroneous data-entry.
