# Supplementary material for: The Impact of Longer Biliopancreatic Limb Length on Weight Loss and Comorbidity Improvement at 5 Years After Primary Roux-en-Y Gastric Bypass Surgery: A Population-Based Matched Cohort Study
Source: Obes Surg. 2024 Jul 9;34(9):3236–45. doi: 10.1007/s11695-024-07267-5 (PMC11349854; doi:10.1007/s11695-024-07267-5)
Supplement: Supplementary file 1 — Supplementary file1 (DOCX 29 KB) [file 11695_2024_7267_MOESM1_ESM.docx]

Supplementary table 1. Data completeness of comorbidity status at the 5-year follow-up moment, calculated over the number of patients that had the comorbidity preoperatively.

|  | Registered comorbidity status at 5 years (%) | |
| --- | --- | --- |
|  | Short BPL  ( < 100 cm) | Long BPL  ( ≥ 100 cm) |
| Diabetes mellitus | 79.2 | 84.9 |
| Hypertension | 34.0 | 37.1 |
| Dyslipidemia | 47.2 | 50.3 |
| OSAS | 29.9 | 27.3 |
| GERD | 16.4 | 23.3 |
| Musculoskeletal pain | 22.4 | 21.9 |

BPL = biliopancreatic limb, cm = centimeter, OSAS = obstructive sleep apnea syndrome, GERD = gastro-esophageal reflux disease.

Supplementary table 2. Baseline characteristics of patients with at least 1-year follow-up data available, before and after propensity score matching

|  | Before matching | | P-value | SMD | After matching | | SMD |
| --- | --- | --- | --- | --- | --- | --- | --- |
|  | BPL < 100 cm | BPL ≥ 100 cm |  |  | BPL < 100 cm | BPL ≥ 100 cm |  |
| n | 15,295 | 13,258 |  |  | 11,518 | 11,518 |  |
|  |  |  |  |  |  |  |  |
| Age (median [range]) | 46 [18, 72] | 47 [18, 73] | <0.01 | 0.13 | 46 [18, 72] | 47 [18, 73] | 0.08 |
| BMI (median (range)), kg/m^2^ | 41.5 [30.2, 75.9] | 41.6 [30.6, 87.1] | 0.06 | 0.03 | 41.5 [30.2, 75.9] | 41.6 [30.6, 87.1] | 0.03 |
| Sex (n, %) |  |  |  |  |  |  |  |
| male | 2,846 (18.6) | 2,567 (19.4) | 0.11 | 0.02 | 2,221 (19.3) | 2,281 (19.8) | 0.01 |
| female | 12,449 (81.4) | 10,691 (80.6) |  |  | 9,297 (80.7) | 9,237 (80.2) |  |
| Last available FU moment (n, %) |  |  |  |  |  |  |  |
| 1-year | 4,150 (27.1) | 4,248 (32.0) | <0.01 | 0.30 | 3,559 (30.9) | 3,559 (30.9) | <0.01 |
| 2-year | 3,478 (22.7) | 3,719 (28.1) |  |  | 2,997 (26.0) | 2,997 (26.0) |  |
| 3-year | 2,443 (16.0) | 2,357 (17.8) |  |  | 2,064 (17.9) | 2,064 (17.9) |  |
| 4-year | 2,357 (15.4) | 1,713 (12.9) |  |  | 1,677 (14.6) | 1,677 (14.6) |  |
| 5-year | 2,867 (18.7) | 1,221 (9.2) |  |  | 1,221 (10.6) | 1,221 (10.6) |  |
| ASA-score (n, %) |  |  |  |  |  |  |  |
| I | 239 (1.6) | 163 (1.2) | <0.01 | 0.28 | 155 (1.3) | 160 (1.4) | 0.02 |
| II | 8,358 (54.6) | 5,467 (41.2) |  |  | 5,303 (46.0) | 5,213 (45.3) |  |
| III | 6,602 (43.2) | 7,523 (56.7) |  |  | 5,979 (51.9) | 6,051 (52.5) |  |
| IV | 52 (0.3) | 55 (0.4) |  |  | 44 (0.4) | 46 (0.4) |  |
| ‘Unknown’ | 44 (0.3) | 50 (0.4) |  |  | 37 (0.3) | 48 (0.4) |  |
| Diabetes mellitus (n, %) |  |  |  |  |  |  |  |
| *Not present* | 12,279 (80.3) | 10,482 (79.1) | <0.01 | 0.06 | 9,186 (79.8) | 9,074 (78.8) | 0.03 |
| *Present without medication* | 958 (6.3) | 727 (5.5) |  |  | 680 (5.9) | 653 (5.7) |  |
| *Present with medication* | 2,058 (13.5) | 2,049 (15.5) |  |  | 1,652 (14.3) | 1,791 (15.5) |  |
| Hypertension (n, %) |  |  |  |  |  |  |  |
| *Not present* | 10,143 (66.3) | 8,211 (61.9) | <0.01 | 0.09 | 7,440 (64.6) | 7,098 (61.6) | 0.06 |
| *Present without medication* | 1,421 (9.3) | 1,308 (9.9) |  |  | 1,073 (9.3) | 1,172 (10.2) |  |
| *Present with medication* | 3,731 (24.4) | 3,739 (28.2) |  |  | 3,005 (26.1) | 3,248 (28.2) |  |
| Dyslipidemia (n, %) |  |  |  |  |  |  |  |
| *Not present* | 12,213 (79.8) | 10,177 (76.8) | <0.01 | 0.09 | 9,069 (78.7) | 8,906 (77.3) | 0.04 |
| *Present without medication* | 1,376 (9.0) | 1,218 (9.2) |  |  | 1,028 (8.9) | 1,063 (9.2) |  |
| *Present with medication* | 1,706 (11.2) | 1,863 (14.1) |  |  | 1,421 (12.3) | 1,549 (13.4) |  |
| OSAS (n, %) |  |  |  |  |  |  |  |
| *Not present* | 12,468 (81.5) | 11,068 (83.5) | <0.01 | 0.12 | 9,544 (82.9) | 9,504 (82.5) | 0.06 |
| *Present without therapy* | 1,270 (8.3) | 1,274 (9.6) |  |  | 966 (8.4)) | 1,126 (9.8) |  |
| *Present with therapy* | 1,557 (10.2) | 916 (6.9) |  |  | 1,008 (8.8) | 888 (7.7)) |  |
| GERD (n, %) |  |  |  |  |  |  |  |
| *Not present* | 12,981 (84.9) | 10,288 (77.6) | <0.01 | 0.21 | 9,511 (82.6) | 9,324 (81.0) | 0.04 |
| *Present without medication* | 1,088 (7.1) | 1,710 (12.9) |  |  | 978 (8.5) | 1,052 (9.1) |  |
| *Present with medication* | 1,226 (8.0) | 1,260 (9.5) |  |  | 1,029 (8.9) | 1,142 (9.9) |  |
| Musculoskeletal pain (n, %) |  |  |  |  |  |  |  |
| *Not present* | 9,121 (59.6) | 6,278 (47.4) | <0.01 | 0.25 | 6,435 (55.9) | 6,053 (52.6) | 0.07 |
| *Present* | 6,174 (40.4) | 6,980 (52.6) |  |  | 5,083 (44.1) | 5,465 (47.4) |  |
|  |  |  |  |  |  |  |  |
| BPL length (median [IQR]), cm | 60 [50 – 75] | 150 [150 – 150] |  |  | 60 [50 – 75] | 150 [150 – 150] |  |
| AL length (median [IQR]), cm | 150 [150 – 150] | 100 [75 – 100] |  |  | 150 [150 – 150] | 100 [75 – 100] |  |

SMD = standardized mean difference, BPL = biliopancreatic limb, cm = centimeter, n = number of cases, SD = standard deviation, FU = follow-up, ASA = American society of anesthesiologists, OSAS = obstructive sleep apnea syndrome, GERD = gastro-esophageal reflux disease, AL = alimentary limb, IQR = inter-quartile range.

Supplementary table 3. Further subdivision of the long BPL group based on AL length and the impact on outcomes at 5 years in matched patients.

|  | **N** | **Patients achieving the outcome** (n, %) | **BPL length**  (median [IQR]) | **OR** (95% CI) | **P**-value |
| --- | --- | --- | --- | --- | --- |
| **25% TWL** (yes/no, %) |  |  |  |  |  |
| Short BPL | 1,264 | 808 (63.9) | 70 [50 – 75] | *Ref.* |  |
| Long BPL – **short** AL | 1,064 | 721 (67.8) | 150 [150 – 150] | 1.18 (1.00 – 1.40) | 0.06 |
| Long BPL – **long** AL | 200 | 137 (68.5) | 100 [100 – 120] | 1.25 (0.91 – 1.37) | 0.18 |
|  |  |  |  |  |  |
| **Improvement in Diabetes** (%) |  |  |  |  |  |
| Short BPL | 259 | 210 (81.1) | 70 [50 – 75] | *Ref.* |  |
| Long BPL – **short** AL | 233 | 211 (90.6) | 150 [150 – 150] | 2.16 (1.26 – 3.71) | < 0.01* |
| Long BPL – **long** AL | 49 | 44 (89.8) | 100 [100 – 120] | 2.22 (0.83 – 5.95) | 0.11 |
|  |  |  |  |  |  |
| **Improvement in Hypertension** (%) |  |  |  |  |  |
| Short BPL | 425 | 308 (72.5) | 70 [50 – 75] | *Ref.* |  |
| Long BPL – **short** AL | 374 | 296 (79.1) | 150 [150 – 150] | 1.44 (1.04 – 2.00) | 0.03* |
| Long BPL – **long** AL | 75 | 60 (80.0) | 100 [100 – 120] | 1.52 (0.83 – 2.78) | 0.18 |
|  |  |  |  |  |  |
| **Improvement in Dyslipidemia** (%) |  |  |  |  |  |
| Short BPL | 227 | 167 (73.6) | 70 [60 – 75] | *Ref.* |  |
| Long BPL – **short** AL | 196 | 145 (74.0) | 150 [150 – 150] | 1.00 (0.65 – 1.55) | 0.99 |
| Long BPL – **long** AL | 17 | 13 (76.5) | 100 [100 – 120] | 1.22 (0.38 – 3.90) | 0.74 |
|  |  |  |  |  |  |
| **Improvement in OSAS** (%) |  |  |  |  |  |
| Short BPL | 137 | 117 (85.4) | 70 [60 – 75] | *Ref.* |  |
| Long BPL – **short** AL | 125 | 114 (91.2) | 150 [150 – 150] | 1.77 (0.81 – 3.87) | 0.16 |
| Long BPL – **long** AL | 27 | 26 (96.3) | 100 [100 – 120] | 4.48 (0.56 – 35.7) | 0.16 |
|  |  |  |  |  |  |
| **Improvement in GERD** (%) |  |  |  |  |  |
| Short BPL | 70 | 63 (90.0) | 70 [70 – 80] | *Ref.* |  |
| Long BPL – **short** AL | 23 | 15 (65.2) | 150 [150 – 150] | 0.16 (0.05 – 0.55) | < 0.01* |
| Long BPL – **long** AL | 19 | 18 (94.7) | 120 [100 – 150] | 2.15 (0.24 – 19.2) | 0.49 |
|  |  |  |  |  |  |
| **Improvement in MSP** (%) |  |  |  |  |  |
| Short BPL | 378 | 228 (60.3) | 70 [65 – 80] | *Ref.* |  |
| Long BPL – **short** AL | 197 | 108 (54.8) | 150 [150 – 150] | 0.80 (0.56 – 1.13) | 0.21 |
| Long BPL – **long** AL | 49 | 35 (71.4) | 100 [100 – 150] | 1.65 (0.86 – 3.17) | 0.13 |
|  |  |  |  |  |  |

Short AL is defined as ≤ 100cm. Long AL is defined as > 100 cm. Short BPL is defined as < 100 cm. Long BPL is defined as ≥ 100 cm.

N = number of patients with the comorbidity at baseline also having status of the outcome of interest at 5 years after surgery, AL = alimentary limb, BPL = biliopancreatic limb, IQR = inter-quartile range, OR = odds ratio, cm = centimeter, TWL = total weight loss, OSAS = obstructive sleep apnea syndrome, GERD = gastroesophageal reflux disease, MSP = musculoskeletal pain, Ref. = reference group,

* = statistically significant

Supplementary table 4. Further subdivision of the long BPL group based on AL length and the impact on outcomes at the last available follow-up moment (1-5 years) in matched patients.

|  | **N** | **Patients achieving the outcome** (n, %) | **BPL length**  (median [IQR]) | **OR** (95% CI) | **P**-value |
| --- | --- | --- | --- | --- | --- |
| **25% TWL** (yes/no, %) |  |  |  |  |  |
| **Short BPL** | 11,520 | 8,876 (77.0) | 60 [50 – 75] | *Ref.* |  |
| Long BPL – **short** AL | 9,224 | 7,273 (79.9) | 150 [150 – 150] | 1.19 (1.11 – 1.27) | < 0.01* |
| Long BPL – **long** AL | 2,296 | 1,896 (82.6) | 100 [100 – 100] | 1.41 (1.26 – 1.59) | < 0.01* |
|  |  |  |  |  |  |
| **Improvement in Diabetes** (%) |  |  |  |  |  |
| **Short BPL** | 1,844 | 1,579 (85.6) | 70 [50 – 75] | *Ref.* |  |
| Long BPL – **short** AL | 1,628 | 1,522 (93.5) | 150 [150 – 150] | 2.36 (1.86 – 2.99) | < 0.01* |
| Long BPL – **long** AL | 352 | 318 (90.3) | 100 [100 – 100] | 1.58 (1.08 – 2.31) | 0.02* |
|  |  |  |  |  |  |
| **Improvement in Hypertension** (%) |  |  |  |  |  |
| **Short BPL** | 3,300 | 2,487 (75.4) | 70 [50 – 75] | *Ref.* |  |
| Long BPL – **short** AL | 2,726 | 2,205 (80.9) | 150 [150 – 150] | 1.38 (1.22 – 1.56) | < 0.01* |
| Long BPL – **long** AL | 784 | 604 (77.0) | 100 [100 – 100] | 1.10 (0.91 – 1.32) | 0.32 |
|  |  |  |  |  |  |
| **Improvement in Dyslipidemia** (%) |  |  |  |  |  |
| **Short BPL** | 1,857 | 1,282 (69.0) | 70 [50 – 75] | *Ref.* |  |
| Long BPL – **short** AL | 1,306 | 931 (71.3) | 150 [150 – 150] | 1.12 (0.96 – 1.31) | 0.15 |
| Long BPL – **long** AL | 616 | 472 (76.6) | 100 [100 – 100] | 1.47 (1.19 – 1.82) | < 0.01* |
|  |  |  |  |  |  |
| **Improvement in OSAS** (%) |  |  |  |  |  |
| **Short BPL** | 1,396 | 1,145 (82.0) | 70 [50 – 75] | *Ref.* |  |
| Long BPL – **short** AL | 1,060 | 954 (90.0) | 150 [150 – 150] | 1.93 (1.51 – 2.46) | < 0.01* |
| Long BPL – **long** AL | 494 | 441 (89.3) | 100 [100 – 100] | 1.85 (1.35 – 2.54) | < 0.01* |
|  |  |  |  |  |  |
| **Improvement in GERD** (%) |  |  |  |  |  |
| **Short BPL** | 937 | 832 (88.8) | 70 [60 – 80] | *Ref.* |  |
| Long BPL – **short** AL | 506 | 391 (77.3) | 150 [150 – 150] | 0.43 (0.32 – 0.58) | < 0.01* |
| Long BPL – **long** AL | 361 | 333 (92.2) | 100 [100 – 100] | 1.50 (0.97 – 2.32) | 0.07 |
|  |  |  |  |  |  |
| **Improvement in MSP** (%) |  |  |  |  |  |
| **Short BPL** | 3,162 | 2,276 (72.0) | 70 [60 – 80] | *Ref.* |  |
| Long BPL – **short** AL | 2,264 | 1,570 (69.3) | 150 [150 – 150] | 0.89 (0.79 – 1.00) | 0.05 |
| Long BPL – **long** AL | 1,156 | 903 (78.1) | 100 [100 – 100] | 1.39 (1.18 – 1.63) | < 0.01* |
|  |  |  |  |  |  |

Short AL is defined as ≤ 100cm. Long AL is defined as > 100 cm. Short BPL is defined as < 100 cm. Long BPL is defined as ≥ 100 cm.

N = number of patients with the comorbidity at baseline also having status of the outcome of interest at 5 years after surgery, AL = alimentary limb, BPL = biliopancreatic limb, IQR = inter-quartile range, OR = odds ratio, cm = centimeter, TWL = total weight loss, OSAS = obstructive sleep apnea syndrome, GERD = gastroesophageal reflux disease, MSP = musculoskeletal pain, Ref. = reference group.
